# Can artificial intelligence–derived coronary atherosclerotic characteristics using CCTA/CACS predict the future onset of atrial fibrillation?

**DOI:** 10.1093/ehjimp/qyae098

**Published:** 2024-09-23

**Authors:** Andrew Chiou, Melody Hermel, Christina Rodriguez Ruiz, Alexander van Rosendael, Tim Burton, Francesca Calicchio, Samantha Bagsic, Eric Hu, Elizabeth Epstein, Casey Joye, Shawn Newlander, Michael Salerno, Sanjeev P Bhavnani, Austin Robinson, Jorge Gonzalez, George E Wesbey

**Affiliations:** Division of Cardiovascular Disease, Scripps Clinic, 9898 Genesee Avenue, AMP 400, La Jolla, CA 92037, USA; Division of Cardiovascular Disease, Scripps Health & United Medical Doctors, La Jolla, CA, USA; Department of Cardiology, Memorial Care Long Beach Medical Center, Long Beach, CA, USA; Department of Cardiology, Leiden University Medical Center, Leiden, The Netherlands; Department of Research & Development, Biostatistics, Scripps Health, La Jolla, CA, USA; Department of Research & Development, Biostatistics, Scripps Health, La Jolla, CA, USA; Department of Research & Development, Biostatistics, Scripps Health, La Jolla, CA, USA; Department of Research & Development, Biostatistics, Scripps Health, La Jolla, CA, USA; Division of Cardiovascular Disease, Scripps Clinic, 9898 Genesee Avenue, AMP 400, La Jolla, CA 92037, USA; Department of Biological Science, Northwestern University, Evanston, IL, USA; Department of Medical Physics, Scripps Health, La Jolla, CA, USA; Department of Cardiovascular Disease, Stanford University, Palo Alto, CA, USA; Division of Cardiovascular Disease, Scripps Clinic, 9898 Genesee Avenue, AMP 400, La Jolla, CA 92037, USA; Division of Cardiovascular Disease, Scripps Clinic, 9898 Genesee Avenue, AMP 400, La Jolla, CA 92037, USA; Division of Cardiovascular Disease & Radiology, Scripps Clinic, La Jolla, CA, USA; Division of Cardiovascular Disease & Radiology, Scripps Clinic, La Jolla, CA, USA

## Introduction

Atrial fibrillation (AF) is the most common arrhythmia and can lead to atherothrombotic vascular events, poor quality of life, and death, so early detection is key. Patients with history of coronary artery disease (CAD) have a higher risk of developing AF, and patients with history of AF have a higher risk of myocardial infarction, suggesting a link in underlying pathophysiology.^1^ High coronary calcium scores (CACS) have been shown to be independently predictive of AF development.^2^ However, despite this connection, it remains unclear which patients affected by CAD will develop AF. Coronary computed tomography angiography (CCTA) analysis of atherosclerotic plaque morphology and characteristics to predict AF development has not been studied. In the present study, we evaluate the association between CCTA coronary atherosclerotic characteristics and the development of AF.

## Methods

This was a retrospective single-centre case-control study. The Scripps Epic database was queried from 1 April 2017 through 30 November 2020 for all adult patients with prior CCTA. These were stable patients with suspected but without known CAD based on abnormal stress testing or suspected CAD symptoms. New-onset AF was identified and adjudicated for each case by a team of experts. Patients with prior mitral valve surgery, postoperative AF, or any occurrence of AF prior to CCTA were excluded. Potential controls were identified as those who were alive at follow-up, at least 18 years of age, and did not have previous catheterization, coronary artery bypass grafting, or percutaneous coronary intervention. Potential controls were selected 1:1 by nearest neighbour propensity matching on the criteria of age, sex, smoker status, hypertension, hyperlipidaemia, and diabetes. Propensity matching was conducted using the package MatchIt version 4.5.2 using R. v. 4.0.3. The study population consisted of 47 patients who experienced new AF and 47 propensity score–matched control individuals.

Anonymized Digital Imaging and Communications in Medicine CCTA images were sent to the CLEERLY laboratory to perform Food and Drug Administration (FDA)–approved machine learning analysis of vessel, lumen, and compositional plaque volume (cubic millimetre) using a series of validated convolutional neural network models. Training, testing, and outputs of algorithms have been previously reported.^3^ The lab was blinded to AF status. Specific coronary atherosclerotic characteristics analysed were as follows: two feature-positive high-risk plaque features (2FPP), total plaque volume, calcified plaque volume (CPV), non-CPV (NCPV), low-density non-CPV (LAPV), CAD-RADS grade, percentage diameter and area stenosis, length (millimetre), and lumen volume. Left atrial volume indices (LAVI) from CCTA were measured by the Total Segmentator plugin to 3D Slicer.^4^ The Total Segmentator plugin to 3D Slicer software was validated in a separate cohort of clinically referred cardiac CT patients (*n* = 63) who received LAVI measures by an FDA-approved commercial software (Visage).^4^ The regression equation resulted in a Pearson *r*^2^ correlation coefficient of *r*^2^ = 0.91 (*P* < 0.01).

## Results

**Figure 1.**
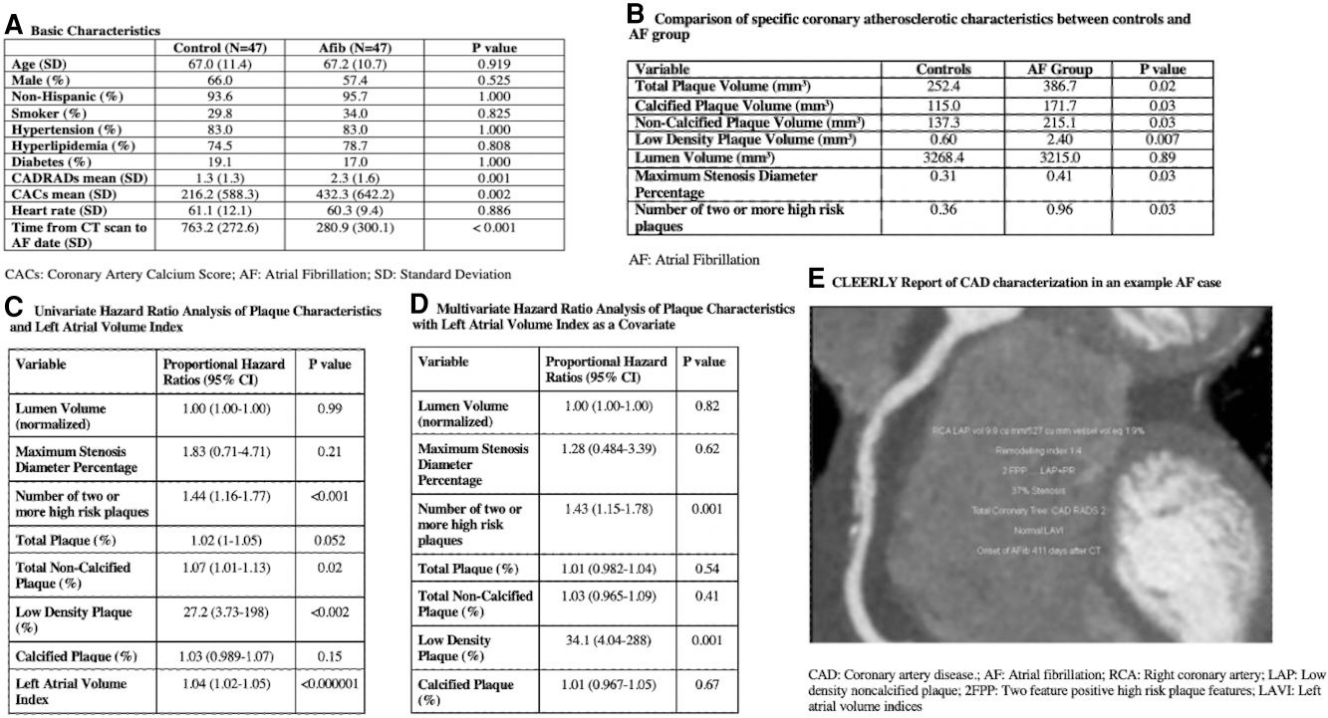
Basic characteristics of the study population and analysis of AI-derived coronary plaque characteristics comparing the atrial fibrillation and control group.

For the study population, the basic characteristics are highlighted in *Figure 1A*. The mean CAD-RADS score as determined by CCTA was 1.8. The mean CAC score was 325, with the AF group showing a significantly higher mean CACS compared to the control group (432.3 vs. 216.2; *P* = 0.002). The mean duration from CCTA to new AF diagnosis for the study patients was 281 ± 300 days. Mean LAVI was 50.5 mL/m^2^.

Patients with new-onset AF were associated with a higher LAVI (56.0 mL/m^2^ vs. 43.5 mL/m^2^ in controls) with a proportional hazard ratio (HR) (95% confidence interval) of 1.04 (1.02–1.05) (*P* < 0.000001). CLEERLY measures in AF and controls and the univariable and multivariate HR analysis of plaque characteristics are presented in *Figure 1*.

## Discussion

To our knowledge, this is the first study that utilizes artificial intelligence (AI)–derived assessment of CCTA to delineate coronary atherosclerotic plaque characteristics associated with individuals who develop new-onset AF (*Figure 1B*). A higher LAVI was found to be associated with the development of AF, similar to previous studies that show an enlarged LA size to be an independent predictor of AF.^1^ In addition, the presence of 2FPP, total NCPV, and LAPV was shown to be associated with a greater risk of new-onset AF (*Figure 1C*). When accounting for LAVI as a covariate, the LAPV HR for AF was 34.1 (4.04–288) (*P* < 0.001) and for 2FPP 1.43 (1.15–1.78) (*P* < 0.001) (*Figure 1D*). This finding is consistent with the previous studies in which atherosclerosis in patients including the presence and severity of carotid plaques has been previously shown to be an independent risk factor for new-onset AF.^5^ It has been hypothesized that the reduction of blood flow to the sinus node and atrial tissue due to atherosclerosis can impact the electrical impulse towards atrial contraction. This reduction of blood flow can also lead to the formation of fibrosis and microscopic scarring of the atrium, further reducing the electrical conduction, resulting in re-entry mechanisms and AF.^6^ Identifying these plaque characteristics may provide incentive for implantable loop recorder use for detection of occult AF. Larger prospective studies could test the generalizability of these findings.

### Limitations

The small sample size of this single-centre study resulted in limited statistical power and wide 95% confidence limits. The retrospective nature of this study also limits our ability to assess causality. There is potential that the control population may have episodic undetected AF, which may cause bias.

## Conclusions

Using AI-derived assessment of CCTA, coronary atherosclerotic plaque characteristics including the presence of 2FPP, total NCPV, and LAPV were shown to be predictive of new-onset AF. This is the first study to show this association and to highlight the predictive possibility of using CCTA to delineate individuals who will develop AF in the future.

## Consent

The Scripps IRB, in its capacity as a privacy board, determined that the current project may use or disclose protected health information. The conditions for waiving the need to obtain individual authorization met the regulations described in US 45 CFR 164.512.

## Data Availability

The data underlying this article are available in the article.

## Lead author biography

Dr Andrew Chiou is a cardiology fellow currently at Scripps Clinic in San Diego, CA, USA. Dr Chiou earned his Bachelor of Science degree at Duke University, and his academic interests paved the way for him to pursue his Doctor of Medicine degree at the University of Nebraska Medical Center. His postgraduate training commenced with an internal medicine residency at the University of California, Davis Medical Center, prior to entering cardiology fellowship. Dr Chiou is committed towards research focusing on the diagnosis and evaluation of ischaemic disease. He is interested in a career in Interventional and Structural Cardiology.

## References

[1] Vaziri SM, Larson MG, Benjamin EJ, Levy D. Echocardiographic predictors of nonrheumatic atrial fibrillation. The Framingham Heart Study. Circulation 1994;89:724–30. 8313561 10.1161/01.cir.89.2.724

[2] O'Neal WT, Efird JT, Dawood FZ, Yeboah J, Alonso A, Heckbert SR et al Coronary artery calcium and risk of atrial fibrillation (from the multi-ethnic study of atherosclerosis). Am J Cardiol 2014;114:1707–12. 25282316 10.1016/j.amjcard.2014.09.005 PMC4253067

[3] Griffin WF, Choi AD, Riess JS, Marques H, Chang HJ, Choi JH et al AI evaluation of stenosis on coronary CTA, comparison with quantitative coronary angiography and fractional flow reserve: a CREDENCE trial substudy. JACC Cardiovasc Imaging 2023;16:193–205. 35183478 10.1016/j.jcmg.2021.10.020

[4] Wasserthal J, Breit HC, Meyer MT, Pradella M, Hinck D, Sauter AW et al TotalSegmentator: robust segmentation of 104 anatomic structures in CT images. Radiol Artif Intell 2023;5:e230024. 37795137 10.1148/ryai.230024 PMC10546353

[5] Heeringa J, van der Kuip DA, Hofman A, Kors JA, van Rooij FJ, Lip GY et al Subclinical atherosclerosis and risk of atrial fibrillation: the Rotterdam study. Arch Intern Med 2007;167:382–7. 17325300 10.1001/archinte.167.4.382

[6] Cox JL, Canavan TE, Schuessler RB, Cain ME, Lindsay BD, Stone C et al The surgical treatment of atrial fibrillation. II. Intraoperative electrophysiologic mapping and description of the electrophysiologic basis of atrial flutter and atrial fibrillation. J Thorac Cardiovasc Surg 1991;101:406–26. 1999934

